# Study protocol for a randomised, double-blind, placebo-controlled 12-week pilot phase II trial of Sailuotong (SLT) for cognitive function in older adults with mild cognitive impairment

**DOI:** 10.1186/s13063-018-2912-0

**Published:** 2018-09-25

**Authors:** Genevieve Z. Steiner, Alan Bensoussan, Jianxun Liu, Mark I. Hohenberg, Dennis H. Chang

**Affiliations:** 1 NICM Health Research Institute and Translational Health Research Institute (THRI), Western Sydney University, Locked Bag 1797, Penrith, NSW 2751 Australia; 2 NICM Health Research Institute, Western Sydney University, Locked Bag 1797, Penrith, NSW 2751 Australia; 3 Xiyuan Hospital, China Academy of Chinese Medical Sciences, Chaoyang District, Beijing, People’s Republic of China; 4 School of Medicine, Western Sydney University, Locked Bag 1797, Penrith, NSW 2751 Australia

**Keywords:** Mild cognitive impairment (MCI), Sailuotong (SLT), Electroencephalography (EEG), Herbal medicine, Neuropsychology, Protocol

## Abstract

**Background:**

Mild cognitive impairment (MCI) is a syndrome characterised by a decline in cognition but relatively intact activities of daily living. People with MCI have an increased risk of developing dementia, and MCI is often referred to as a transitional stage between healthy ageing and dementia. Currently, there are no pharmaceutical therapies approved by the US Federal Drug Administration for MCI. Randomised controlled trials on the two major classes of anti-dementia pharmaceuticals, cholinesterase inhibitors and glutamate receptor antagonists, have produced poor results in MCI cohorts. There is a need to test and evaluate new and promising treatments for MCI that target multiple aspects of the syndrome’s multi-faceted pathophysiology. The primary aim of this study is to evaluate the efficacy of 12 weeks of treatment with a standardised herbal formula, Sailuotong (SLT), compared to placebo, on cognition in older adults with MCI. Secondary aims are to assess SLT’s mechanisms of action via electroencephalography (EEG), autonomic function, brain blood flow, and inflammation, as well as its safety in this cohort.

**Methods/design:**

The target cohort for this trial is community-dwelling older adults over the age of 60 years who meet the National Institute of Aging-Alzheimer’s Association working group core clinical criteria for MCI due to Alzheimer’s disease. Eighty participants will be recruited and randomly allocated via a permuted block strategy at a 1:1 ratio to either the treatment or placebo group. The co-primary cognitive outcome measures are Logical Memory Story A delayed recall (episodic memory), Letter Number Sequencing (perceptual processing speed), and both the Trail Making Test and Rey Complex Figure Test (executive function). Secondary outcome measures are EEG activity, autonomic function (via electrocardiogram, skin conductance, and peripheral pulse pressure), brain blood flow (via common carotid artery ultrasound), and serum concentrations of inflammatory cytokines. Analyses will be performed blind to group allocation.

**Discussion:**

This study is a 12-week, randomised, double-blind, placebo-controlled trial. Primary and secondary outcome measures will be compared between treatment and placebo groups at baseline and endpoint. Data from this pilot study will inform a larger, more highly powered clinical trial if the findings are positive.

**Trial registration:**

Australian New Zealand Clinical Trials Registry (ANZCTR), ACTRN12617000371392 Registered on 10 March 2017.

**Electronic supplementary material:**

The online version of this article (10.1186/s13063-018-2912-0) contains supplementary material, which is available to authorized users.

## Background

### Overview

Mild cognitive impairment (MCI) causes a slight but noticeable decline in cognitive abilities and is conceptualised as a transitional stage between healthy ageing and dementia. It is estimated that up to 35% of Australians aged 70 and older have MCI [1], and 15% of those individuals will go on to develop dementia within a year [2]. Currently, there are no approved treatment options for MCI, and evidence on anti-dementia pharmaceuticals does not support their use in people with MCI [3–5]. Sailuotong (SLT) is a novel, standardised herbal medicine preparation that has been specifically designed to target the complex pathophysiologies of vascular dementia and Alzheimer’s disease. This project will evaluate the efficacy, safety, and the mechanisms of action SLT for MCI in a 12-week randomised, double-blind, placebo-controlled pilot trial.

### MCI characteristics

MCI is a heterogeneous syndrome that is associated with an increased risk of dementia. People with MCI demonstrate objective cognitive decline but have relatively intact activities of daily living. Not all people with MCI will go on to develop dementia, and considerable efforts have been made to establish clinical phenotypes that may represent the prodromal phase for different types of dementia and to provide clinical utility in determining risk [6, 7]. MCI can be classified into clinical subtypes across two broad categories:

- Amnestic MCI, where some form of memory impairment is present
- Non-amnestic MCI, where there is impairment in non-memory domain(s).

These classifications can be further divided into single- or multiple-domain impairments [8], where one or more cognitive domains are affected. The most common subtype, amnestic MCI, is considered the prodromal phase for Alzheimer’s disease [9], the most frequent cause of dementia. Furthermore, population studies also suggest that individuals with multiple-domain amnestic subtype MCI are most at risk of developing dementia [2].

There is no consensus on one set of diagnostic guidelines for MCI. Clinical categorisations of MCI include the Diagnostic and Statistical Manual of Mental Disorders, 4th Edition (DSM-IV) classification of Cognitive Disorder Not Otherwise Specified [10], the Petersen criteria [6], the joint National Institute on Aging-Alzheimer’s Association (NIA-AA) criteria for MCI due to Alzheimer’s disease [11], and the recent addition to the DSM-V: Mild Neurocognitive Disorder [12]. No clear cut-off scores or instruments are defined in any of these guidelines, making these criteria difficult to apply in a research setting. Instead, it is recommended that a clinical diagnosis of amnestic MCI is made (taking into account a range of evidence including both subjective and objective decline) [6, 8], as outlined in the core clinical criteria for MCI due to Alzheimer’s disease from the NIA-AA working groups guidelines [11].

### Limitations of current treatment strategies for MCI

Multiple mechanisms are thought to be involved in the pathophysiology of MCI (which is thought to be due to the early signs of Alzheimer’s disease), including chronic inflammation, anti-oxidant depletion, neurotransmitter dysfunctions (e.g. cholinergic hypofunction), and cerebral hypoxia, that can play a role in the biochemical cascade leading to increased amyloid-β production, mitochondrial dysfunction, and free radical generation [13–17].

Currently, there are no approved therapeutic agents for MCI by the US Federal Drug Administration (FDA) that may manage symptoms in the short term or prevent/slow the conversion of MCI to Alzheimer’s disease in the long term [18]. Standard clinical management of MCI involves monitoring of risk factors and cognitive decline. Pharmaceutical intervention is possible once a diagnosis of dementia is certain, and it then focuses on symptomatic management and decreasing the rate of deterioration. Cholinesterase inhibitors (donepezil, galantamine, and rivastigmine) and glutamate receptor antagonists (memantine) are the most common classes of pharmaceuticals used for Alzheimer’s disease; however, evidence of their efficacy and safety for MCI is poor and inconsistent.

The very first large-scale donepezil trial on MCI (in combination with vitamin E) observed a slower rate of progression to Alzheimer’s disease during the first 12 months, but at the 36 month follow-up, there was no difference in conversion rate between treatment groups and placebo [3]. A very large 2-year trial showed that treatment with galantamine did not differ from placebo in the conversion rate of MCI to dementia [4]. Although there were some small treatment-related improvements in cognition after 12–24 months, there was also a significantly higher mortality rate, suggesting that galantamine is not safe for individuals with MCI. A 4-year trial of rivastigmine for MCI showed no difference in dementia conversion rates and cognitive performance between treatment and placebo, with side effects including nausea, vomiting, diarrhoea, and dizziness also being reported in the treatment group [5]. There are no robust, fully powered trials on memantine for MCI [19, 20]; however, it has been suggested that there is limited evidence to support such an attempt [21]. The findings from the early trials outlined above have also been confirmed by a meta-analysis which found no effect of donepezil, rivastigmine, galantamine, or memantine on cognition or function in patients with MCI, but rather found an increased risk of nausea, diarrhoea, and vomiting compared to placebo [18].

In summary, the existing evidence to support the efficacy of anti-dementia pharmaceuticals in MCI is relatively weak. The benefits of these interventions for MCI require validation, particularly in light of the long-term management of symptoms required for patients and the risk of adverse side effects [18]. Furthermore, conventional pharmaceutical treatments typically act on a single pharmacological target and do not address the multiple pathological components of MCI as a whole. Taken together, these findings indicate that new treatments should achieve all of the following:

- Address the pathophysiology of MCI through a multi-target approach
- Improve and/or stabilise cognitive deficits
- Safely manage symptoms in the long term.

### Multi-system/multi-target approach of an herbal formula for MCI

The use of herbal medicine for ageing-related disorders and longevity was documented in the literature more than 2000 years ago in ancient China. Combination therapy underpins the philosophy of Chinese herbal medicine, where patients are typically treated with multi-herbal formulations. Evidence suggests that a number of medicinal herbs, including *Ginkgo biloba* (ginkgo), *Panax ginseng* (ginseng), and *Crocus sativus* (saffron), may aid in the treatment of cognitive impairment [22]. This led to the development of a three-herb formula, Sailuotong (SLT; previously known as Wei Nao Kang, WNK), by a combined team based at Xiyuan Hospital, China Academy of Chinese Medical Sciences, China and the National Institute of Complementary Medicine (NICM), Western Sydney University, Australia. The SLT formula consists of specific dosages of standardised ginkgo, ginseng, and saffron extracts, designed for the management of cognitive impairments. The data from a series of preclinical and clinical studies show that SLT can affect multiple therapeutic targets associated with MCI and dementia [23].

### SLT formulation background

SLT represents a new generation of herbal formulations for which the chemical and pharmacological profiles have been clearly defined using analytical chemistry, pharmacognosy, and pharmacology techniques. The preclinical research contributing to the development and standardisation of SLT [23–30], the mechanisms of action of the three herbs [23, 31–38], and findings from clinical studies [22, 39–48] have all been described thoroughly elsewhere and summarised in a recent review by Chang et al. [23]. Table 1 details the multiple pharmacological targets that the active components in SLT address relevant to the pathophysiology of MCI.

**Table 1:** Multi-target mechanisms underlying pharmacological effects of SLT components

Pharmacological effects relevant to MCI	Ginseng	Ginkgo	Saffron
Anti-inflammatory	✓	✓	✓
Anti-oxidant	✓	✓	✓
Anti-apoptotic	✓	✓	✓
Increasing cerebral blood flow		✓	
Anti-platelet aggregation		✓	
Antagonising amyloid-β accumulation	✓		
Enhancement of cholinergic function	✓		
Anti-depressant/anti-anxiety		✓	✓

### Rationale for the current project

Dementia is the single greatest cause of disability in Australians aged 65 years and older, is the second leading cause of death in Australia [49], and currently has no cure. There are more than 413,106 Australians living with dementia, and this figure is expected to rise to more than 1,100,890 by 2056 without medical breakthrough [50]. MCI, a syndrome associated with a significantly increased risk of dementia, is a growing health concern for seniors, with up to 80% of patients with MCI developing dementia within 6 years [51].

Evidence on the efficacy and safety supporting the use of anti-dementia drugs in MCI is lacking. Furthermore, these treatments have focused on single therapeutic targets such as cholinesterase or glutamate which do not adequately address the complex and multi-system nature of MCI. SLT, however, consists of multiple bioactive components addressing different pathophysiological causes of cognitive impairments through various pharmacological activities that reduce inflammation, oxidative stress, and apoptosis; inhibit platelet aggregation; and increase blood flow, anti-oxidant capacity, and brain tissue acetylcholine (ACh) concentrations (see Table 1). Preclinical work and pilot clinical trials have provided convincing evidence on the beneficial effects of SLT in various animal models in healthy adults, and in people with vascular dementia. Together, this suggests that SLT can potentially provide a viable intervention for the management of MCI. A clinical trial with a rigorous design is needed to confirm the efficacy of SLT on cognitive function, its mechanisms of action, and its safety in an MCI cohort.

## Methods/design

### Study design, ethics, and setting

The study design is a randomised, double-blind, placebo-controlled trial. Information on the testing schedule and the outcome measures is detailed further below. Ethics approval was requested and approved through the Western Sydney University Human Research Ethics Committee (Approval H11878). The trial was registered with the Australian New Zealand Clinical Trials Registry (ACTRN12617000371392) on 10 March 2017. The current study protocol (version 3) was designed in accordance with Standard Protocol Items: Recommendations for Interventional Trials (SPIRIT) [52] (see Additional file 1) and Good Clinical Practice guidelines (1996). Any changes to the study protocol will be communicated with the study investigative team, the Australian New Zealand Clinical Trials Registry, and the approving ethics committee. The study is being conducted in the NICM Clinical Laboratory and HEADBOX Lab at Western Sydney University, Campbelltown campus.

### Study aims

The primary aim of this project is to evaluate the efficacy of 12 weeks of SLT (compared with placebo) on cognition in people with MCI. The secondary aims of this study are to 1. assess the mechanisms of action of SLT via electroencephalography (EEG), autonomic function, cerebral blood flow, and inflammation, and 2. evaluate the safety of SLT.

### Intervention

#### SLT treatment

The SLT formula is standardised by 10 bioactive components of the three herbal extracts from *Panax ginseng*, *Ginkgo biloba*, and *Crocus sativa*. A range of in vivo pharmacological studies have been undertaken to test the bioactivity and determine the optimal ratio of the three individual herbal extracts. Each SLT capsule contains a 45-mg standardised mixture of 20.46 mg ginsenosides from *Panax ginseng*, 20.46 mg total ginkgo flavone glycosides from *Ginkgo biloba*, and 4.09 mg crocins from *Crocus sativa*. The SLT preparations have been manufactured in a Good Manufacturing Practice facility certified by the Australian Therapeutic Goods Administration. The dose is 180 mg (4 × 45-mg capsules, 2 in the morning and 2 at night) of SLT per day for 12 weeks.

#### Placebo

The placebo consists of 4 capsules per day (2 each morning and night), containing an inert substance (starch filler, silica coating, with taste and colouring added) matched for the colour, taste, and smell of SLT.

### Recruitment, consent, and reimbursement

A number of avenues are being pursued for recruitment. Advertisements will be placed in local papers in surrounding areas; flyers will be placed around local community centres and residential aged care facilities as well as Western Sydney University campuses. Internet advertisements will be placed on several websites including those for Western Sydney University, NICM, Alzheimer’s Australia, the Dementia Research Foundation, and the Dementia Collaborative Research Centres. Advertisements will also be placed in university newsletters and at other external organisations such as Illawarra Retirement Trust (IRT) where possible. The study will also be promoted through social media including Twitter and Facebook. A link to the study’s screening page will be included in all online recruitment pathways. Participants will be directed to complete the online screening form, and their contact details will be collected. Local general practitioner (GP)/specialist clinics will be contacted and encouraged to identify and refer suitable MCI patients to this study. Potentially eligible participants from other studies in our laboratory will also be invited to participate in this trial.

Written information on the study will be provided, and participants will have the opportunity to verbally discuss the study with the research team. At a face-to-face screening, the participant and his informant will each sign an informed consent form.

Participants will be offered reimbursement to cover travel expenses for their study visits: up to $30/visit will be provided. Each participant will complete 6 visits (1 × screening, 2 × baseline, 1 × midpoint, 2 × endpoint), totalling up to $180. Parking will be provided free of charge at Western Sydney University’s Campbelltown campus.

### Inclusion/exclusion criteria

Participants will be assessed against the following inclusion criteria:

- ≥ 60 years of age
- No diagnosis of dementia
- Confirmed diagnosis of MCI due to Alzheimer’s disease core clinical criteria according to the NIA-AA working group guidelines [11]
- No severe depression: scoring ≤19 on the 30-item Geriatric Depression Scale (GDS)
- Agreement to provide informed consent.

They will also be assessed for the following exclusion criteria:

- Diagnosed psychiatric disorders including dissociative disorder, obsessive-compulsive disorder, personality disorders, schizophrenia, bipolar disorder
- History of drug and alcohol dependence or substance-related disorders
- History of seizures
- Head trauma with loss of consciousness
- Left-handedness (for EEG testing)
- Allergic to at least one ingredient of SLT
- Current use of ginkgo, ginseng, or saffron
  - ○ Participants taking these herbal medicines will be asked to cease use for an 8-week washout period prior to participation in the trial
- History of severe renal and hepatic disorders.

### Sample size

There are no similar studies using SLT for the proposed study duration (12 weeks) in an MCI cohort. Thus, we selected a well-designed study that used the same primary outcome measure and a similar cohort with a 16-week intervention. That study also provided effect sizes at midpoint (8 weeks) [53]. Craft et al. [53] demonstrated significantly improved delayed recall scores on the Logical Memory Story A subtest of the Wechsler Memory Scale, 4th Edition (WMS-IV) following 8 weeks of 20 IU insulin: a treatment group × time interaction, *p* = 0.020, Cohen’s *f* = 0.36.

We utilised this effect size to conduct an a priori sample size calculation based on the Logical Memory Story A subtest of the WMS-IV. To detect an effect size of Cohen’s *f* = 0.36 at α = 0.05 and 80% power, 63 participants across two groups are required. Allowing for a 20% dropout, this means that 76 participants (38 per group) are required; we rounded this to 80 participants to facilitate a permuted block randomisation strategy. Note also that because this study is a pilot efficacy study (phase II), a larger sample size to reach a higher degree of statistical power is not required.

### Assessments and testing schedule

**Fig. 1:**
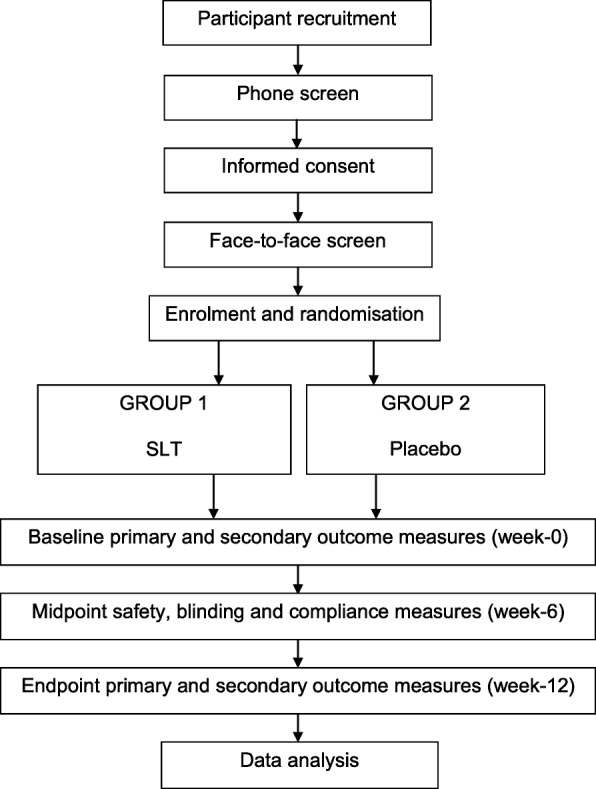
Schematic of participant flow

**Fig. 2:**
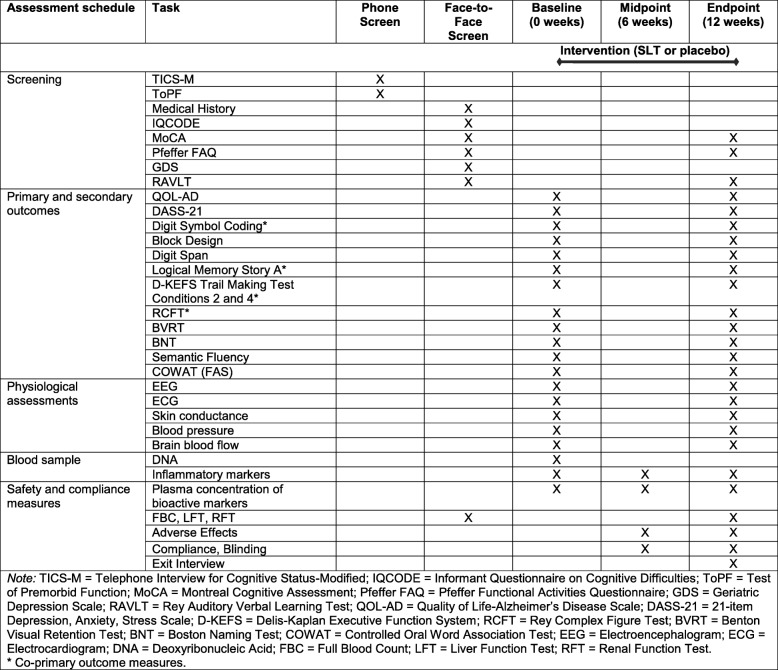
Schedule of interventions and assessments at screening (phone and face-to-face), baseline, midpoint, and endpoint sessions

A schematic of the trial protocol is detailed in Fig. 1. Eligible participants will be screened via telephone (45 min) and invited to attend a face-to-face screening session (1.5–2 h) where informed consent and verification of safety to participate (via commercial laboratory blood test) will be obtained. Once enrolled in the study, participants will be randomly allocated to either the treatment or placebo condition. Two testing sessions (cognitive and physiological) will be conducted at baseline and at endpoint over 2 separate days (3–4 h each); the midpoint assessment will be conducted on a single day (30 min). The schedule of assessments is detailed in Fig. 2.

### Screening assessment

#### Phone screen

Participants will undergo an initial telephone interview assessing them against the study’s inclusion and exclusion criteria and estimating cognitive function and decline using the 13-item modified Telephone Interview for Cognitive Status (TICS-M) and Test of Premorbid Functioning (ToPF), respectively.

#### Face-to-face screen

After written informed consent is obtained from both the participant and her informant, the participant’s medical history and demographics will be taken with a semi-structured clinical interview, and the participant will be assessed against the NIA-AA core clinical criteria for MCI due to Alzheimer’s disease [11] using the Informant Questionnaire on Cognitive Decline in the Elderly (IQCODE) [54], the Montreal Cognitive Assessment (MoCA) [55], the Pfeffer Functional Activities Questionnaire (FAQ), the GDS [56, 57], and the Rey Auditory Verbal Learning Test (RAVLT). The various screening instruments, their purpose, and administration method are summarised in Table 2.

**Table 2:** List of items used during screening, their purpose, and administration method

Task	Purpose	Administration method
TICS-M	Screen for cognitive informant	Test: participant and informant
ToPF	Level of functioning prior to cognitive decline	Survey: participant
Pfeffer FAQ	Activities of daily living	Survey: informant
IQCODE	Informant questionnaire to corroborate cognitive difficulties	Survey: informant
MoCA	Screen for MCI	Test: participant
RAVLT	Objective memory impairment	Test: participant
Medical history	General health and functioning	Interview: participant and informant
GDS	Depression screen	Survey: participant

*Abbreviations: TICS-M* Telephone Interview for Cognitive Status-Modified, *ToPF* Test of Premorbid Functioning, *Pfeffer FAQ* Pfeffer Functional Activities Questionnaire, *IQCODE* Informant Questionnaire on Cognitive Decline in the Elderly, *MoCA* Montreal Cognitive Assessment, *RAVLT* Rey Auditory Verbal Learning Test, *GDS* Geriatric Depression Scale

### Primary outcome measures

There are four co-primary outcome measures:

- Logical Memory Story A
- Digit Symbol Coding
- Delis-Kaplan Executive Function System (D-KEFS) Trail Making Test Condition 4
- Rey Complex Figure Test.

People living with multiple-domain amnestic subtype MCI^1^ showing deficits in episodic memory, executive function, and perceptual processing speed are particularly at risk of progressing to Alzheimer’s disease [58]. Episodic memory is the most reliable predictor of this conversion [58]; thus, an appropriate and widely used episodic memory measure (Logical Memory Story A) was selected for the sample size calculation. The other three co-primary outcome measures were selected due to their relevance in testing the other domains: Digit Symbol Coding measures perceptual processing speed, and the D-KEFS Trail Making Test Condition 4 and the Rey Complex Figure Test measure executive function.

### Secondary outcome measures

#### Cognitive assessments

A comprehensive neuropsychological test battery, based on the Sydney Memory and Ageing Study (detailed in Brodaty et al. [2]), measuring the five cognitive domains of memory, executive function, attention/processing speed, language, and visuospatial abilities and allowing for comparison with normative demographic-adjusted data was selected. The compendium of tests is detailed in Table 2.

#### Mood and quality of life

The 21-item Depression, Anxiety, Stress Scale (DASS-21) and the Quality of Life in Alzheimer’s Disease (QOL-AD) scale will be used to assess mood and quality of life, respectively.

#### Physiological assessments

Mechanisms of action of SLT will be assessed using central and autonomic nervous system measures of EEG, electrocardiogram (ECG), skin conductance, peripheral pulse pressure, and brain blood flow (common carotid artery ultrasound) at rest and in response to audio/visual stimuli to index changes in psychophysiological processes.

#### Inflammatory markers

Blood samples will be collected from all participants to further assess the mechanisms of action of SLT via change in serum inflammatory marker concentrations, including homocysteine [59], α1-antichymotrypsin [60, 61], interleukin-6 (IL-6) [60], IL-1β [62], and tumour necrosis factor alpha (TNF-α) [62]. DNA will also be extracted from the blood samples, and genetic variants identified in genome-wide association studies will be investigated due to their association with Alzheimer’s disease risk; subgroup analyses (e.g. APOE-E4 carriers/non-carriers) will also be conducted.

### Randomisation and allocation concealment

A university staff member, external to the research team, conducted computerised random sequence allocation using a unique random number generator in Microsoft Excel. A permuted block randomisation strategy was used with an allocation ratio of 1:1. Allocation was concealed using batch numbers generated with the unique random number generator by the same staff member; these were sent directly to the manufacturer. After eligibility is confirmed, participants are allocated to the next numbered box of product by a member of the research team. This is a double-blind trial, so both participants and the research team are blinded to allocation. Furthermore, all data analyses will be conducted blinded to treatment allocation.

### Statistical analyses and data management

The study will largely employ a mixed-model design with a between-subjects factor of group (treatment vs. placebo) and within-subjects factor of time (baseline vs. endpoint). Analyses of variance (ANOVAs) will be conducted to assess differences in continuous outcome measures between the two groups. All tests will be one tailed, α = 0.05. As there are only two testing points for the primary outcome measures (baseline and endpoint), data from any participants failing to complete follow-up will not be included in the final analysis.

All data will be stored on a secure server with two back-up copies on external hard drives. Paper-based forms will be digitised and the original copies stored in locked filing cabinets in secure rooms that require swipe card access at Western Sydney University Campbelltown campus. All participants are de-identified upon randomisation and referred to on all forms with a participant ID. A password-protected spreadsheet stored on the secure server links participant names to ID codes to all for re-identification to occur if required. As this is a relatively small investigator-initiated pilot trial, a data monitoring committee and auditing process are not required.

### Compliance, success of blinding, safety, and adverse events

After baseline measurements, the first 6 weeks of medication will be dispensed. The second 6 weeks of medication will be dispensed at the midpoint of the study. An additional week of medication (7 weeks total) will be provided at both baseline and midpoint in case there is an unforeseen delay in attending the follow-up appointments. Participants will be required to return any unused medication at midpoint and endpoint; this will be used to determine compliance. The number of returned capsules will be counted by a university staff member external to the research team to further reduce the potential of unblinding. The blood samples collected at baseline, midpoint, and endpoint will also be used in a separate pharmacokinetics study to assess the plasma concentrations of bioactive components of SLT. Eight SLT markers will be assessed: ginsenosides Rg1, Re, Rb1, and Rc and ginkgolides A, B, C, and bilobalide.

At the study midpoint and endpoint, participants will be asked to which treatment condition they think that they have been allocated. Responses of SLT, placebo, or unknown will be recorded. Responses will be checked against the condition allocated at the end of the trial to assess success of blinding. An exit interview will be conducted upon study completion to ascertain further information about participants’ experiences during the trial, e.g. information on health-related expectations, factors influencing compliance/adherence, and any impacts that the trial medication might have had on quality of life. Participants who exit the study early will be contacted via telephone and requested to complete the exit interview.

To monitor safety, participants will be referred to a local commercial laboratory (Laverty) at screening and endpoint. Standard blood safety tests (full blood count and liver and renal function tests) will be carried out. If participants receive an abnormal blood safety test result at baseline, they will not be enrolled in the study, and they will be encouraged to follow up their results with their regular health professional. Adverse events will be closely monitored throughout the course of the study. In the case of a serious adverse event, if the study drug is suspected as a potential cause and identifying treatment allocation is essential to the participant receiving appropriate medical care, unblinding of that participant only will occur. This will be done by the university staff member external to the research team who generated the computerised random sequence allocation.

## Discussion

SLT has shown efficacy in improving cognition in people with vascular dementia and in enhancing neuronal activity and cerebral perfusion in healthy adults and in seniors with vascular dementia, respectively. Given that preclinical and pilot clinical trials have also shown SLT to be safe and well tolerated, and because it addresses multiple mechanisms implicated in the pathology of MCI, it should be evaluated in this cohort.

This study has some limitations. First, although an a priori sample size calculation has been determined, the study still has a relatively low power (80%) to detect an effect. Having said this, the project is a pilot study — the first of its kind in an MCI cohort — and the outcomes will inform the design of a high-powered conversion study to test whether SLT can prevent or delay the development of dementia. Second, participant burden is quite high during the physiological assessment battery due to the number of measures and the length of the tasks. However, this is necessary given the trial’s secondary aim to assess the mechanisms of action of SLT in people with MCI. Scheduled breaks have been put in place to minimise participant fatigue.

MCI is a heterogeneous syndrome with complex pathophysiologies. Current efforts to address the cognitive deficits in MCI using single-target pharmaceuticals, such as cholinesterase inhibitors, have yielded poor and inconsistent results, highlighting the need for new and innovative strategies. This randomised controlled trial focuses on SLT, a potential treatment that has been shown to address MCI’s multi-faceted pathophysiology in preclinical studies and has yielded positive results from pilot clinical trials in healthy adults and people with vascular dementia. The outcomes of this trial will inform the development of future interventional research involving people with MCI, such as a large-scale conversion study.

### Trial status

The trial began recruitment on 3 April 2017 and is currently ongoing.

## Additional file

Additional file 1: SPIRIT 2013 checklist: recommended items to address in a clinical trial protocol and related documents. (DOC 123 kb)

## Abbreviations

ACh: Acetylcholine
ADAS-Cog: Alzheimer’s Disease Assessment Scale-Cognitive subscale
ATP: Adenosine triphosphate
BNT: Boston Naming Test
BVRT: Benton Visual Retention Test
COWAT: Controlled Oral Word Association Test
DASS-21: 21-item Depression, Anxiety, Stress Scale
D-KEFS: Delis-Kaplan Executive Function System
DNA: Deoxyribonucleic acid
DSM-IV: Diagnostic and Statistical Manual of Mental Disorders, 4th Edition
ECG: Electrocardiogram
EEG: Electroencephalography
FBC: Full blood count
FDA: Federal Drug Administration
GDS: Geriatric Depression Scale
GP: General practitioner
IQCODE: Informant Questionnaire on Cognitive Decline in the Elderly
LFT: Liver function test
MCI: Mild cognitive impairment
MoCA: Montreal Cognitive Assessment
NIA-AA: National Institute on Aging-Alzheimer’s Association
Pfeffer FAQ: Pfeffer Functional Activities Questionnaire
QOL-AD: Quality of Life in Alzheimer’s Disease (scale)
RAVLT: Rey Auditory Verbal Learning Test
RCFT: Rey Complex Figure Test
RFT: Renal function test
SLT: Sailuotong
SOD: Superoxide dismutase
TICS-M: Telephone Interview for Cognitive Status-Modified
ToPF: Test of Premorbid Functioning
WMS-IV: Wechsler Memory Scale, 4th Edition
WNK: Wei Nao Kang

## Footnotes

1 Memory deficit plus impairment in one or more other cognitive domain(s).

## References

[1] Sachdev PS (2010). The Sydney Memory and Ageing Study (MAS): methodology and baseline medical and neuropsychiatric characteristics of an elderly epidemiological non-demented cohort of Australians aged 70-90 years. Int Psychogeriatr.

[2] Brodaty H (2013). Mild cognitive impairment in a community sample: the Sydney Memory and Ageing Study. Alzheimers Dement.

[3] Petersen RC (2005). Vitamin E and donepezil for the treatment of mild cognitive impairment. N Engl J Med.

[4] Winblad B (2008). Safety and efficacy of galantamine in subjects with mild cognitive impairment. Neurology.

[5] Feldman HH (2007). Effect of rivastigmine on delay to diagnosis of Alzheimer’s disease from mild cognitive impairment: the InDDEx study. Lancet Neurol.

[6] Petersen RC (1999). Mild cognitive impairment: clinical characterization and outcome. Arch Neurol.

[7] Petersen RC (2001). Current concepts in mild cognitive impairment. Arch Neurol.

[8] Petersen RC (2004). Mild cognitive impairment as a diagnostic entity. J Intern Med.

[9] Espinosa A (2013). A longitudinal follow-up of 550 mild cognitive impairment patients: evidence for large conversion to dementia rates and detection of major risk factors involved. J Alzheimers Dis.

[10] American Psychiatric Association (1994). Diagnostic and statistical manual of mental disorders.

[11] Albert MS (2011). The diagnosis of mild cognitive impairment due to Alzheimer's disease: recommendations from the National Institute on Aging-Alzheimer's Association workgroups on diagnostic guidelines for Alzheimer's disease. Alzheimers Dement.

[12] Sachs-Ericsson N, Blazer DG (2015). The new DSM-5 diagnosis of mild neurocognitive disorder and its relation to research in mild cognitive impairment. Aging Ment Health.

[13] Bishop NA, Lu T, Yankner BA (2010). Neural mechanisms of ageing and cognitive decline. Nature.

[14] Van der Schyf CJ (2006). Multifunctional neuroprotective drugs targeting monoamine oxidase inhibition, iron chelation, adenosine receptors, and cholinergic and glutamatergic action for neurodegenerative diseases. Expert Opin Investig Drugs.

[15] Cahill-Smith S, Li JM (2014). Oxidative stress, redox signalling and endothelial dysfunction in ageing-related neurodegenerative diseases: a role of NADPH oxidase 2. Br J Clin Pharmacol.

[16] Frank-Cannon TC (2009). Does neuroinflammation fan the flame in neurodegenerative diseases?. Mol Neurodegener.

[17] Bartus RT (2000). On neurodegenerative diseases, models, and treatment strategies: lessons learned and lessons forgotten a generation following the cholinergic hypothesis. Exp Neurol.

[18] Tricco AC (2013). Efficacy and safety of cognitive enhancers for patients with mild cognitive impairment: a systematic review and meta-analysis. CMAJ.

[19] Ferris S (2007). A double-blind, placebo-controlled trial of memantine in age-associated memory impairment (memantine in AAMI). Int J Geriatr Psychiatry.

[20] Peters O (2012). A combination of galantamine and memantine modifies cognitive function in subjects with amnestic MCI. J Nutr Health Aging.

[21] McClendon MJ (2009). Memantine and acetylcholinesterase inhibitor treatment in cases of CDR 0.5 or questionable impairment. J Alzheimer’s Dis.

[22] Dennis C, Ben C, Rong L (2013). Chinese medicine used to treat dementia. Advances in natural medicines, nutraceuticals and neurocognition.

[23] Chang D (2016). Herbal medicine for the treatment of vascular dementia: an overview of scientific evidence. Evid Based Complement Alternat Med.

[24] Xu L (2007). Effects of Weinaokang (WNK) on dysmensia in mice models. Pharmacol Clin.

[25] Cong WH, Liu JX, Xu L (2007). Effects of extracts of ginseng and Ginkgo biloba on hippocampal acetylcholine and monoamines in PDAP-pV717I transgenic mice. Zhongguo Zhong Xi Yi Jie He Za Zhi.

[26] Xu L (2008). Effects of Weinaokang capsule on intracephalic cholinergic system and capability of scavenging free radicas in chronic cerebral hypoperfusion rats. Zhongguo Zhong Yao Za Zhi.

[27] Cong W-h (2012). Herbal extracts combination (WNK) prevents decline in spatial learning and memory in APP/PS1 mice through improvement of hippocampal Aβ plaque formation, histopathology, and ultrastructure. Evid Based Complement Alternat Med.

[28] Liu JX (2004). Effect of combination of extracts of ginseng and ginkgo biloba on acetylcholine in amyloid beta-protein-treated rats determined by an improved HPLC. Acta Pharmacol Sin.

[29] Zheng YQ (2007). Effects of crocin on reperfusion-induced oxidative/nitrative injury to cerebral microvessels after global cerebral ischemia. Brain Res.

[30] Zhang Y (2014). Pharmacokinetics and brain distribution of ginsenosides after administration of sailuotong. Zhongguo Zhong Yao Za Zhi.

[31] Radad K (2006). Use of ginseng in medicine with emphasis on neurodegenerative disorders. J Pharmacol Sci.

[32] Chu S (2014). Ginsenoside Rg5 improves cognitive dysfunction and beta-amyloid deposition in STZ-induced memory impaired rats via attenuating neuroinflammatory responses. Int Immunopharmacol.

[33] Scholey AB, Kennedy DO (2002). Acute, dose-dependent cognitive effects of Ginkgo biloba, Panax ginseng and their combination in healthy young volunteers: differential interactions with cognitive demand. Hum Psychopharmacol.

[34] Chan PC, Xia Q, Fu PP (2007). Ginkgo biloba leave extract: biological, medicinal, and toxicological effects. J Environ Sci Health C Environ Carcinog Ecotoxicol Rev.

[35] Smith JV, Luo Y (2004). Studies on molecular mechanisms of Ginkgo biloba extract. Appl Microbiol Biotechnol.

[36] Amin B (2014). Attenuation of oxidative stress, inflammation and apoptosis by ethanolic and aqueous extracts of Crocus sativus L. stigma after chronic constriction injury of rats. An Acad Bras Cienc.

[37] Abe K, Saito H (2000). Effects of saffron extract and its constituent crocin on learning behaviour and long-term potentiation. Phytother Res.

[38] Hosseinzadeh H, Sadeghnia HR (2005). Safranal, a constituent of Crocus sativus (saffron), attenuated cerebral ischemia induced oxidative damage in rat hippocampus. J Pharm Pharm Sci.

[39] Gauthier S, Schlaefke S (2014). Efficacy and tolerability of Ginkgo biloba extract EGb 761(R) in dementia: a systematic review and meta-analysis of randomized placebo-controlled trials. Clin Interv Aging.

[40] Tan MS (2015). Efficacy and adverse effects of ginkgo biloba for cognitive impairment and dementia: a systematic review and meta-analysis. J Alzheimers Dis.

[41] DeKosky ST (2008). Ginkgo biloba for prevention of dementia: a randomized controlled trial. JAMA.

[42] Reay JL, Kennedy DO, Scholey AB (2006). Effects of Panax ginseng, consumed with and without glucose, on blood glucose levels and cognitive performance during sustained 'mentally demanding' tasks. J Psychopharmacol.

[43] Heo JH (2008). An open-label trial of Korean red ginseng as an adjuvant treatment for cognitive impairment in patients with Alzheimer's disease. Eur J Neurol.

[44] Lee ST (2008). Panax ginseng enhances cognitive performance in Alzheimer disease. Alzheimer Dis Assoc Disord.

[45] Kennedy DO, Scholey AB, Wesnes KA (2001). Differential, dose dependent changes in cognitive performance following acute administration of a Ginkgo biloba/Panax ginseng combination to healthy young volunteers. Nutr Neurosci.

[46] Yakoot M, Salem A, Helmy S (2013). Effect of Memo(R), a natural formula combination, on Mini-Mental State Examination scores in patients with mild cognitive impairment. Clin Interv Aging.

[47] Steiner GZ (2016). The effect of Sailuotong (SLT) on neurocognitive and cardiovascular function in healthy adults: a randomised, double-blind, placebo controlled crossover pilot trial. BMC Complement Altern Med.

[48] Jia J (2018). Efficacy and safety of the compound Chinese medicine SaiLuoTong in vascular dementia: a randomized clinical trial. Alzheimer's Dement.

[49] Australian Institute of Health and Welfare (AIHW). Leading Causes of Death; 2013. https://www.aihw.gov.au/reports/life-expectancy-death/deaths-in-australia/contents/leading-causes-of-death.

[50] National Centre for Social and Economic Modelling (NATSEM) (2017). Economic cost of dementia in Australia 2016-2056.

[51] Morris JC (2001). Mild cognitive impairment represents early-stage Alzheimer disease. Arch Neurol.

[52] Chan A-W (2013). SPIRIT 2013 explanation and elaboration: guidance for protocols of clinical trials. BMJ.

[53] Craft S (2012). Intranasal insulin therapy for Alzheimer disease and amnestic mild cognitive impairment. Arch Neurol.

[54] Jorm AF (2004). The Informant Questionnaire on Cognitive Decline in the Elderly (IQCODE): a review. Int Psychogeriatr.

[55] Nasreddine ZS (2005). The Montreal Cognitive Assessment, MoCA: a brief screening tool for mild cognitive impairment. J Am Geriatr Soc.

[56] Yesavage JA (1982). Development and validation of a geriatric depression screening scale: a preliminary report. J Psychiatr Res.

[57] Debruyne H (2009). Is the geriatric depression scale a reliable screening tool for depressive symptoms in elderly patients with cognitive impairment?. Int J Geriatr Psychiatry.

[58] Backman L (2005). Cognitive impairment in preclinical Alzheimer's disease: a meta-analysis. Neuropsychology.

[59] Seshadri S (2002). Plasma homocysteine as a risk factor for dementia and Alzheimer's disease. N Engl J Med.

[60] Engelhart MJ (2004). Inflammatory proteins in plasma and the risk of dementia: the Rotterdam study. Arch Neurol.

[61] Licastro F (1995). Increased serum α1-antichymotrypsin in patients with probable Alzheimer's disease: an acute phase reactant without the peripheral acute phase response. J Neuroimmunol.

[62] Swardfager W (2010). A meta-analysis of cytokines in Alzheimer's disease. Biol Psychiatry.

